# Long-term stress levels are synchronized in dogs and their owners

**DOI:** 10.1038/s41598-019-43851-x

**Published:** 2019-06-06

**Authors:** Ann-Sofie Sundman, Enya Van Poucke, Ann-Charlotte Svensson Holm, Åshild Faresjö, Elvar Theodorsson, Per Jensen, Lina S. V. Roth

**Affiliations:** 1 IFM Biology, AVIAN Behavioural Genomics and Physiology group, Linköping University, 581 83 Linköping, Sweden; 2 Department of Medical and Health Sciences, Linköping University, 581 85 Linköping, Sweden; 3 Department of Clinical and Experimental Medicine, Linköping University, 581 85 Linköping, Sweden

**Keywords:** Animal physiology, Animal behaviour

## Abstract

This study reveals, for the first time, an interspecific synchronization in long-term stress levels. Previously, acute stress, has been shown to be highly contagious both among humans and between individuals of other species. Here, long-term stress synchronization in dogs and their owners was investigated. We studied 58 dog-human dyads and analyzed their hair cortisol concentrations (HCC) at two separate occasions, reflecting levels during previous summer and winter months. The personality traits of both dogs and their owners were determined through owner-completed Dog Personality Questionnaire (DPQ) and human Big Five Inventory (BFI) surveys. In addition, the dogs’ activity levels were continuously monitored with a remote cloud-based activity collar for one week. Shetland sheepdogs (N = 33) and border collies (N = 25), balanced for sex, participated, and both pet dogs and actively competing dogs (agility and obedience) were included to represent different lifestyles. The results showed significant interspecies correlations in long-term stress where human HCC from both summer and winter samplings correlated strongly with dog HCC (summer: N = 57, χ^2^ = 23.697, P < 0.001, β = 0.235; winter: N = 55, χ^2^ = 13.796, P < 0.001, β = 0.027). Interestingly, the dogs’ activity levels did not affect HCC, nor did the amount of training sessions per week, showing that the HCC levels were not related to general physical activity. Additionally, there was a seasonal effect in HCC. However, although dogs’ personalities had little effects on their HCC, the human personality traits neuroticism, conscientiousness, and openness significantly affected dog HCC. Hence, we suggest that dogs, to a great extent, mirror the stress level of their owners.

## Introduction

Emotional contagion, the mirroring of emotional or arousal states between individuals, is commonly seen among group-living species^1^, for example as a synchronization of acute stress responses^2,3^. Social animals, spending time together, are continuously exposed to shared stressors which could affect different individuals similarly^4^. Also, stress has been suggested to be highly contagious within individuals of the same species^5,6^. For example, students have been shown to have high cortisol concentrations when they have teachers who experience high levels of stress^6^, and social prairie voles (*Microtus ochrogaster*) show correlating stress levels with a partner who has previously been exposed to a stressor^5^.

Not only does emotional contagion occur within a species, it has also been shown to occur between species, for example between dogs and humans^4,7^, making the dog-human dyad a good model for studying this. Dogs and humans are two social species that share a unique interspecies relationship as a result of living in close association for at least 15,000 years^8^. Today, most dogs live as companion animals, sharing both environment and everyday life of their human owners. Stress contagion can occur between the two species as measured by cognitive performance^7^, but it has also been shown that short-term cortisol responses are related within dog-human dyads during the performance of dog sports^9^.

The facts that short-term stress seems contagious between dogs and owners and that dogs share their owners’ everyday life could lead to an interspecific long-term stress hormone synchronization within the dyad. Whereas acute cortisol levels can be assessed in matrices such as blood and saliva^10^, a promising matrix to assess long-term cortisol concentration in is hair^11^. As the hair grows, cortisol from the blood is gradually incorporated, in effect forming a retrospective calendar of cortisol concentrations. Hence, hair cortisol concentrations (HCC) allow us to study possible long-term stress synchronisation. Previously, synchrony of hair cortisol concentrations has been found between mothers and their children^12,13^.

Certain characteristics of the dog-human dyad have been suggested to influence stress responsiveness. Many dog owners actively train and compete with their dogs in disciplines such as agility and obedience, and previous research shows that training and competing may influence both dogs’ human-directed social behavior^14^ and hair cortisol levels^15^. Training also seems to increase owners’ emotional closeness to their dogs^16^, and thus affects the characteristics of the dyad. Moreover, saliva cortisol concentrations of dog-human dyads were affected by owner personality traits and the gender-sex combination of the dyad^17^. Interestingly, it seems that owner characteristics are more influential on the dog than vice versa^16,17^.

In the present study, our aim was to investigate interspecific contagiousness of long-term stress levels by using dogs and their owners. We studied 58 dog-human dyads and analyzed hair cortisol concentrations (HCC) in both dogs and their female owners at two separate occasions, reflecting cortisol levels during summer and winter months. Since cortisol secretion can be affected by physical activity^18^, the dogs’ activity levels were continuously monitored with a remote cloud-based activity collar for one week and the owners reported daily routines. Dogs were of two different breeds (Shetland sheepdog N = 33 and border collie N = 25), balanced for sex, and we included both pet dogs and actively competing dogs (agility and obedience) to account for different lifestyles. In addition to cortisol synchronization, we investigated the possible influence of personality traits of both owners and dogs on the long-term stress level of the dog-human dyad.

## Results

### Effects of human HCC, breed, sex and lifestyle on dog HCC

**Figure 1:**
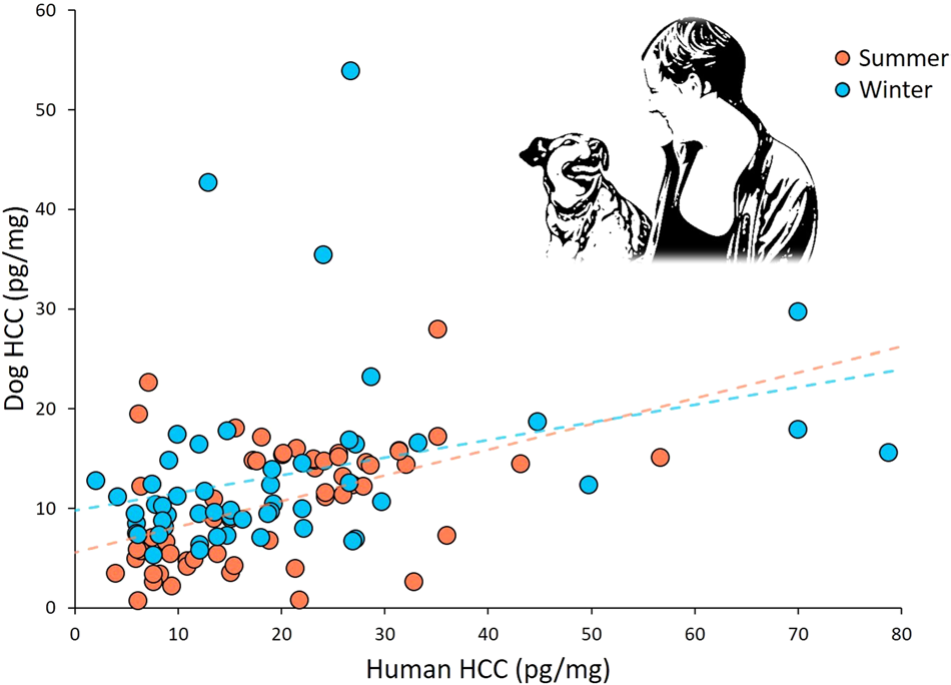
The hair cortisol concentrations (HCC) of dogs and their owners was synchronized at two separate sampling occasions reflecting summer (red dots; N = 57) and winter (blue dots; N = 55) months. Dotted lines show linear fitted lines for respective sampling occasion.

**Figure 2:**
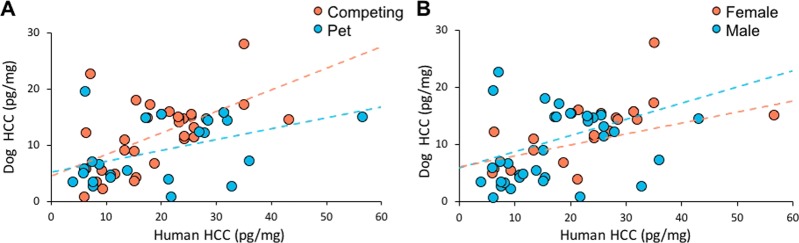
The hair cortisol concentration (HCC) synchronization of dogs and their owners was moderated by lifestyle (**A** competing dogs red, pet dogs blue) and sex of the dog (**B** females red, males blue). Dotted lines show linear fitted lines for lifestyle and sex of the dog.

**Figure 3:**
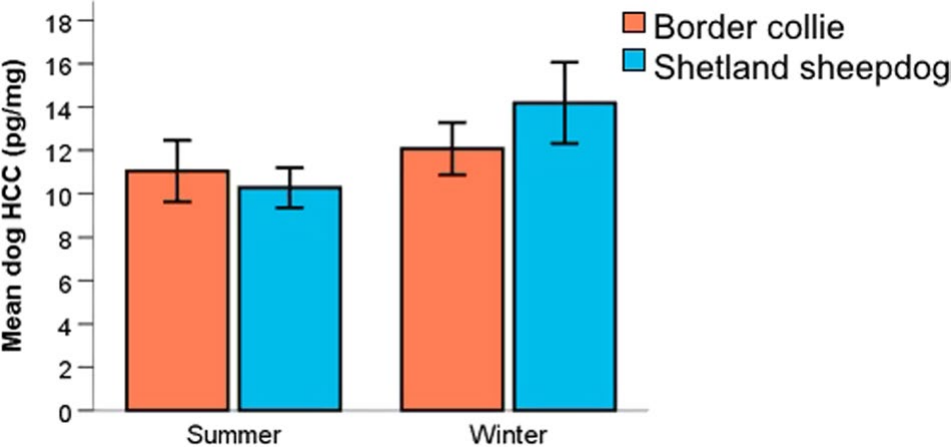
Hair cortisol concentrations (HCC) from the winter sample differed between border collies (BC) and Shetland sheepdogs (SS). Error bars indicate 1 SEM.

The effect of human HCC on dog HCC was analyzed with a generalized linear model (GLM) for both winter and summer samples. Breed, sex, and lifestyle (pet or competing) were included in the models. Human HCC had a significant effect on dog HCC for both summer (Fig. 1; N = 57, χ^2^ = 23.697, P < 0.001, β = 0.235) and winter (N = 55, χ^2^ = 13.796, P < 0.001, β = 0.027) samples. With an increase in human HCC, there was an increase in dog HCC. Additionally, for the summer samples, we found interactions between human HCC and lifestyle (Fig. 2A; χ^2^ = 6.268, P = 0.012) and between human HCC and dogs’ sex (Fig. 2B; χ^2^ = 5.200, P = 0.023). The HCC of both pet and competing dogs as well as of both males and female dogs had an effect of human HCC, but it was stronger in competing dogs and in female dogs. On winter dog HCC there was an effect of breed (Fig. 3; χ^2^ = 6.451, P = 0.011), and Shetland sheepdogs had a higher HCC than border collies (12.905 ± 1.417 vs. 12.069 ± 1.203; mean ± SEM). Factors such as age of dog and owner, if the dogs had access to a garden, owners’ work status (fulltime, part-time, not working), and whether the dogs lived together with other dogs did not affect HCC and were not included in the models.

### Effects of physical activity

Since cortisol concentrations can be affected by physical activity, we wanted to see if the long-term HCC correlated to dogs’ general activity level. The activity of dogs was continuously monitored with a remote cloud-based activity collar (PetPace™) for one week. Out of these seven days, three weekdays and one weekend day were chosen and thoroughly analyzed. The amount of time the dogs spent in medium to high activity was correlated to dog HCC using Spearman’s rank correlation and no significant correlations were found to either summer HCC (N = 44, r = −0.213, P = 0.165) or winter HCC (N = 43, r = −0.239, P = 0.122). Neither did training amount per week, as reported by the owners, correlate to dog HCC (summer: N = 47, r = −0.142, P = 0.340; winter: N = 45, r = −0.218, P = 0.150).

### Effects of dog and human personality

We were also interested in seeing how both the dogs’ and humans’ personalities affected the dog HCC. By using the validated personality surveys “Dog Personality Questionnaire” (DPQ; Jones 2008) and “The Big Five Inventory” (for the owners) (BFI; Zakrisson 2010) the personalities of both involved parties were investigated. Human personality traits had extensive impact on dog HCC. The score on Neuroticism in the owner had a negative association with dog HCC in both summer (N = 57; χ^2^ = 7.951, P = 0.005, β = −0.364) and winter (χ^2^ = 4.919, P = 0.027, β = −0.026) samples (SI Appendix, S1A). In addition, the scores on Conscientiousness and Openness were positively associated with HCC in winter samples (SI Appendix, S1B,C; N = 55; χ^2^ = 15.852, P < 0.001, β = 0.005 and χ^2^ = 11.440, P = 0.001, β = 0.019, respectively).

There were also interactions between owner personality and sex of the dogs. For summer samples, there was an interaction between dogs’ sex and Openness where HCC among female dogs increased with higher scores on Openness, while HCC decreased among male dogs (χ^2^ = 5.390, P = 0.020). For winter samples, there was an interaction with dogs’ sex for owner traits Agreeableness, Conscientiousness, and Neuroticism. An increase in owner Agreeableness was associated with an increase in HCC in male dogs but not in female dogs (χ^2^ = 4.298, P = 0.038). The HCC of both male and female dogs increased with higher owner Conscientiousness, but the association was stronger in males (χ^2^ = 11.540, P = 0.001). For the trait Neuroticism, dog females’ HCC increased with higher scores for Neuroticism while it decreased for males (χ^2^ = 21.437, P < 0.001). For dog personality, on the other hand, traits had little effect on dog HCC, and the only significance was an interaction between sex of the dog and the trait Responsiveness to training on winter HCC. Female dogs with high scores for this trait had low HCC whereas male dogs with a high score for the trait had high HCC (χ^2^ = 9.144, P = 0.002).

### Seasonal effects on dog HCC

**Figure 4:**
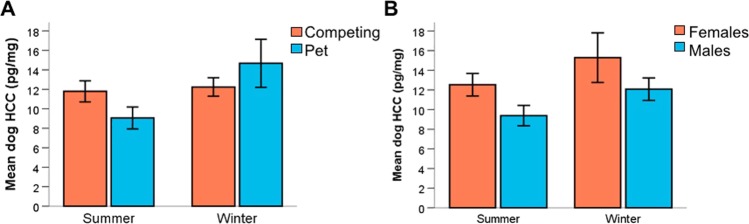
Mean dog hair cortisol concentration (HCC) in (**A**) competing and pet dogs and (**B**) female and male dogs during both summer and winter months. Error bars indicate 1 SEM.

Since hair samples were collected on two separate occasions, we used a generalized linear mixed model to investigate the effect of season and found that dog HCC was higher during winter months compared to summer months (F_1,106_ = 8.706, P = 0.004). There was an interaction between season and lifestyle, and the HCC of pet dogs showed a greater increase in the winter sample than competition dogs (Fig. 4A F_1,106_ = 6.143, P = 0.015). In addition, there was also an effect of dogs’ sex and female dogs showed higher HCC when considering both time points (Fig. 4B; F_1,106_ = 5.270, P = 0.024). There was no seasonal effect on owner HCC.

## Discussion

**Figure 5:**
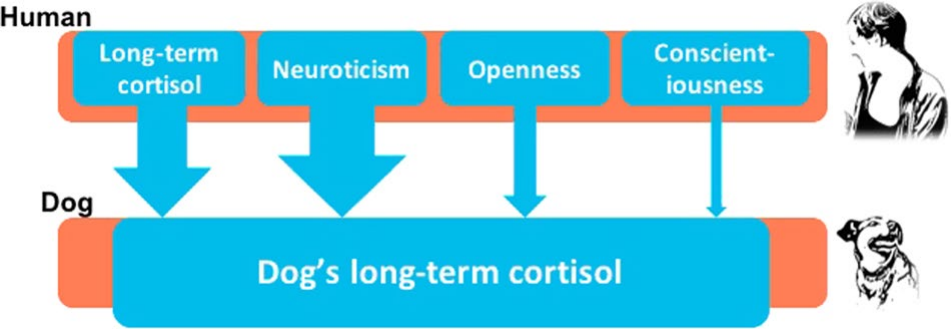
The dog hair cortisol concentration (HCC) was affected by owner HCC, owner personality traits Neuroticism, Openness and Conscientiousness. The thickness of the arrows corresponds to the contribution of each variable to the model (β coefficients; see results).

Through assessment of cortisol concentration in hair of both dogs and their owners, we found an interspecific long-term stress hormone synchronization within the dog-human dyad (Fig. 5). This was observed in two different seasons within one year and was not related to the dogs’ physical activity level. There was also a seasonal effect, and dog HCC was higher during the winter months. Additionally, we show that owner’s personality rather than dog’s personality affects HCC, and therefore suggest that dogs mirror the stress of their owners. Our results are the first demonstration of a long-term synchronization in stress levels between members of two different species.

The evidence of short-term stress contagion within a species is compelling^5^ and Buttner, *et al*.^9^ shows that there is also a cortisol synchronization between species, namely between handlers and their dogs during agility competition. However, this can partly be caused by the mutual physical activity during such a competition and does not necessarily reflect contagious effects of psychological stress. Here, we found synchronized long-term stress levels in dog-human dyads, containing both pet and competing dogs of two different dog breeds, providing further evidence to the strong relationship between humans and dogs. Long-term stress contagion has previously been shown between human mothers and both their infants^13^ and their older children^12^. However, this is the first study on long-term interspecies stress synchronization.

While there was a synchronization for all tested groups of dogs, the synchrony was affected by sex and lifestyle. The HCC of both male and female dogs was synchronized with owner HCC, but the association was stronger in female dogs. Furthermore, in general, female dogs showed a higher cortisol concentration than male dogs. Indeed, studies on other species have previously suggested that, compared to males, females show a higher emotional responsivity. This is found in humans^19^, chimpanzees^20^, and rats^21^, and it has been suggested that the ultimate cause is the different social roles of males and females^22^. Within the human-dog dyad, oxytocin administration in dogs has a pronounced effect on female dogs’ interaction with their owner, and, in addition, an increase in owner oxytocin concentration, but there is no similar effect in males^23^. Thus, both our results and those of Nagasawa, *et al*.^23^ show that dogs’ sex affects hormonal synchronization.

The interaction between lifestyle and human HCC on dog HCC suggests a stronger association in cortisol synchronization among competing dyads than among pet dyads. It may be that competing owners and their dogs spend more time together engaging in the same tasks. Indeed, training may increase emotional closeness as earlier suggested by Meyer and Forkman^16^ and thereby generate a stronger synchronization. Similarly, Roth, *et al*.^15^ found that competing dogs have higher HCC than other dogs. Of course, the difference between competing and pet dogs may not only be the lifestyle in itself, they may also differ in traits not covered within the scope of this study. Certain traits may make a dog more suitable for canine sports that may also affect the stress response.

The HCC could not be related to the dogs’ physical activity obtained by smart collars or the training frequency reported by the owners. We did, however, find characteristics that significantly affected dog HCC. Interestingly, we show that owner personality, or more specifically, the traits Neuroticism, Openness and Conscientiousness, influence long-term cortisol concentrations in the dog. Both Kotrschal, *et al*.^24^ and Schöberl, *et al*.^17^ found that cortisol concentration in morning saliva is lower in dogs with more owners scoring high on Neuroticism. This is in line with our results, and owners that scored high on Neuroticism also had dogs with low HCC. There is some indication that humans scoring high on Neuroticism form a strong attachment bond to their dogs and that these individuals, to a greater extent than others, use their dog as a social supporter whilst also simultaneously functioning as a social supporter for their dog^24^. This, in turn, may lead to a positive modulation of the stress response for both parties.

We also found a positive association between dog HCC and scores on both Openness and Conscientiousness in the owners, but whereas Neuroticism affected HCC from both sampling occasions, Openness and Conscientiousness only had an effect on winter samples. Additionally, similarly to what was reported by Roth, *et al*.^15^, there was a seasonal effect on dog HCC and cortisol was higher during the winter months. This was significant for the Shetland sheepdogs but not for the border collies. It could be speculated that some dogs are more affected by cold winter temperatures, but future studies will need to disentangle seasonal effects to reveal possible causations.

There were significant interactions between personality traits and sex, where HCC in male and female dogs was differently affected by owner personality. Schöberl, *et al*.^17^ found that owner cortisol was lower in women owning male dogs. We only included female owners, but our results are still in line with this observation. Contrary to owner personality, dog personality traits had little effect on dog HCC and consistent with previous studies there was no significant correlations between short-term cortisol concentrations and dog personality^17,25,26^.

Questionnaires are at a risk of being biased by the person completing them and, in this study, both dog and owner personality surveys were completed by the same person. However, both surveys have been validated and the questions are formulated differently in the two, hence, it is unlikely that answering one questionnaire has influenced the owner when completing the other.

The facts that we observe synchronization between dog and human cortisol concentrations and that characteristics of the owner rather than those of the dog are related to dog cortisol levels, make us suggest that it is the dogs that mirror the stress levels of their owners rather than the opposite. This may be relevant from the perspective of the welfare of dogs since stress and related health issues are of great concern in today’s human society. From the human’s point of view, the dog is an important social supporter, has positive effects on learning ability and several health aspects^24,27–29^. From the dog’s point of view, there are indeed several positive effects of the human-dog interaction^30,31^, but our results suggest human-dog matches may be important for the stress levels of dogs.

## Conclusion

Our results show that long-term stress hormone levels were synchronized between dogs and humans, two different species sharing everyday life. This could not be explained by either physical activity or by the amount of training. Since the personality of the owners was significantly related to the HCC of their dogs, we suggest that it is the dogs that mirror the stress levels of their owners rather than the owners responding to the stress in their dogs. To our knowledge, this is the first study to show interspecies synchronization of long-term stress.

## Materials and Methods

### Ethical note

This study was conducted in line with ethical approval from the regional ethical committee for animal experiments (Permit number: 51-13) with the inclusion of human subjects (Permit number: 2017/94-31) in Linköping, Sweden. All methods were performed in accordance with the relevant guidelines and regulations and all dog owners were informed and gave their written consent that they voluntarily participated in the study.

### Study subjects

The participating dyads were recruited through social media and personal contacts and consisted of 33 Shetland sheepdogs (18 females and 15 males) and 25 border collies (13 females and 12 males), and their female owners (Dataset 1). Dogs were regularly walked and all lived indoors with their owners. The dogs were grouped into either companion (15 Shetland sheepdog and 11 border collies) or competing dogs (18 Shetland sheepdogs and 14 border collies) where competing dyads reported that they actively trained and competed in either agility, obedience, or both disciplines. The mean age of competing dogs was 4.7 ± 0.4 (SEM) years and 4.7 ± 0.7 (SEM) years for pet dogs. The mean age of the owners was 46.1 ± 1.7 (SEM).

### Personality and lifestyle surveys

Personalities of the dogs and owners were assessed through owner-completed Dog Personality Questionnaire (DPQ)^32^ and Big Five Inventory (BFI)^33^. The validated DPQ^32^ is a 75-item questionnaire rated on a 5-point Likert scale resulting in five different factors: Activity/Excitability, Responsiveness to training, Aggression towards people, Aggression towards animals, and Fearfulness. The validated BFI^33^ consisted of 44 statements that were rated on a 5-point Likert scale and assessed the personality traits Extraversion, Agreeableness, Conscientiousness, Neuroticism and Openness.

In addition, information on daily routines with the dog, housing, training amount, and competing frequency was collected. The dogs were defined as competing dogs if they actively trained and competed in agility or/and obedience, otherwise they were classified as pet dogs. A dog-owner dyad always shared a certain lifestyle, i.e. competing or pet lifestyle (Dataset 1).

### Hair cortisol analysis

Hair samples were obtained from both owners and their dogs at two separate occasions (September/October 2017 and February 2018). The dog hair was cut from the neck as close to the skin as possible, and the human hair was cut off close to the scalp from the posterior vertex area of the head. The hair was stored at room temperature until cortisol extraction, and radioimmunoassay was performed according to methods described previously^15,34^. Before extraction, approximately 7 mg hair from the three most proximal centimeters was cut in small pieces, weighed exactly for later calculations, frozen in liquid nitrogen for 2 min, and homogenized with a steel ball using a Tissue lyzer II (2 min 23 Hz). Methanol (1 ml) was added to each tube, after which they were put in a tube shaker overnight. On the following day, the samples were centrifuged (23 G, 1 min, 4 °C) and 0.8 mL of the methanol supernatant was pipetted off and lyophilized using a Savant Speed Vac Plus SC210A (1.5 h). The remaining pellets were stored in the refrigerator until further analysis. Finally, the pellets were dissolved in 200 µL RIA buffer. Fifty  µL of this dissolved solution was then taken and 100 µL of primary antibody (Anti-cortisol rabbit antibody) was added. After a 48 h incubation period, 100 µL of radioactive conjugated cortisol was added to each tube, these were then incubated for 24 h. After this incubation, 75 µL of SAC-CEL (Solid phase second anti-rabbit antibody coated cellulose suspension) was added to all samples. The reaction was stopped after 30 min by adding 2 mL of water. The samples were then centrifuged for 15 min (3000 rpm; 4 °C). The water was removed using a decanting tool, and the tubes were placed in a gamma counter (PerkinElmer 2470 Wizard2) which gave measures in CPM and nmol/L, later converted into pg of cortisol per mg of hair. Samples were run in duplicates for validity and the inter-assay coefficient of variability was found to be 12.5, while the intra-assay coefficient of variability was 9.7.

### Long-term activity monitor

Forty-four of the dogs were equipped with a PetPace™ smart collar for seven days during September/October. Out of these seven days three weekdays and one day during the weekend were chosen for further analysis in order to avoid days with missing data points. The PetPace™ is a wireless cloud-based collar that monitors parameters such as activity. The collar collected activity data continuously resulting in activity scores every 2 min. Each data point belonged to the activity categories rest, low, medium, and high, based on PetPace™ algorithm for accelerometer outputs from the collar

### Data analysis

All statistical analyses were performed in IBM® SPSS® software version 25. Generalized linear models (GLMs) were used to analyze effects on dog HCC. Residuals were checked for distribution and normal identity was used as model type for summer dog HCC, and gamma with log link was used for winter dog HCC. The models to investigate the interaction between dog HCC and human HCC consisted of: human HCC (covariate), dogs’ sex (fixed factor), breed (fixed factor), and lifestyle (fixed factor) as main effects, and two-way interactions between human HCC and the fixed factors. The models to investigate the effect of owner and dog personality consisted of: personality traits (covariates) and dogs’ sex (fixed factor), and two-way interactions between personality traits and dogs’ sex. For analysis of seasonal effects on dog HCC from both sampling occasions we used a generalized linear mixed model (GLMM) with season (summer/winter) as repeated measure. Dog HCC was used as dependent variable and model type was gamma with log link. Season, dogs’ sex, lifestyle, and breed were used as fixed effects, and two-way interactions between season and the other factors were included. Akaike’s information criterion was used to determine best model fit. The correlations between dog HCC and activity measurements (PetPace™ data and training amount) were tested with a Spearman’s rank correlation test. For GLMs and GLMM, the final models are presented in (SI Appendix, Tabel S1).

## Supplementary information

Supplementary files
