# Author Correction: Long-term stress levels are synchronized in dogs and their owners

**DOI:** 10.1038/s41598-020-74204-8

**Published:** 2020-10-08

**Authors:** Ann-Sofie Sundman, Enya Van Poucke, Ann-Charlotte Svensson Holm, Åshild Faresjö, Elvar Theodorsson, Per Jensen, Lina S. V. Roth

**Affiliations:** 1 IFM Biology, AVIAN Behavioural Genomics and Physiology Group, Linköping University, 581 83 Linköping, Sweden; 2 Department of Medical and Health Sciences, Linköping University, 581 85 Linköping, Sweden; 3 Department of Clinical and Experimental Medicine, Linköping University, 581 85 Linköping, Sweden

Correction to: *Scientific Reports* 10.1038/s41598-019-43851-x, published online 06 June 2019

This Article contains errors.

In the Results section,

“On winter dog HCC there was an effect of breed (Fig. 3; χ^2^ = 6.451, *P* = 0.011), and Shetland sheepdogs had a higher HCC than border collies (12.905 ± 1.417 vs. 12.069 ± 1.203; mean ± SEM).”

should read:

“On winter dog HCC there was an effect of breed (Fig. 3; χ^2^ = 6.451, *P* = 0.011), and Shetland sheepdogs had a higher HCC than border collies (14.185 ± 1.877 vs. 12.069 ± 1.203; mean ± SEM).”

Additionally, in Figure 2B, the trend line colours are incorrect and should be reversed. The correct Figure 2 appears below as Figure 1.

**Figure 1:**
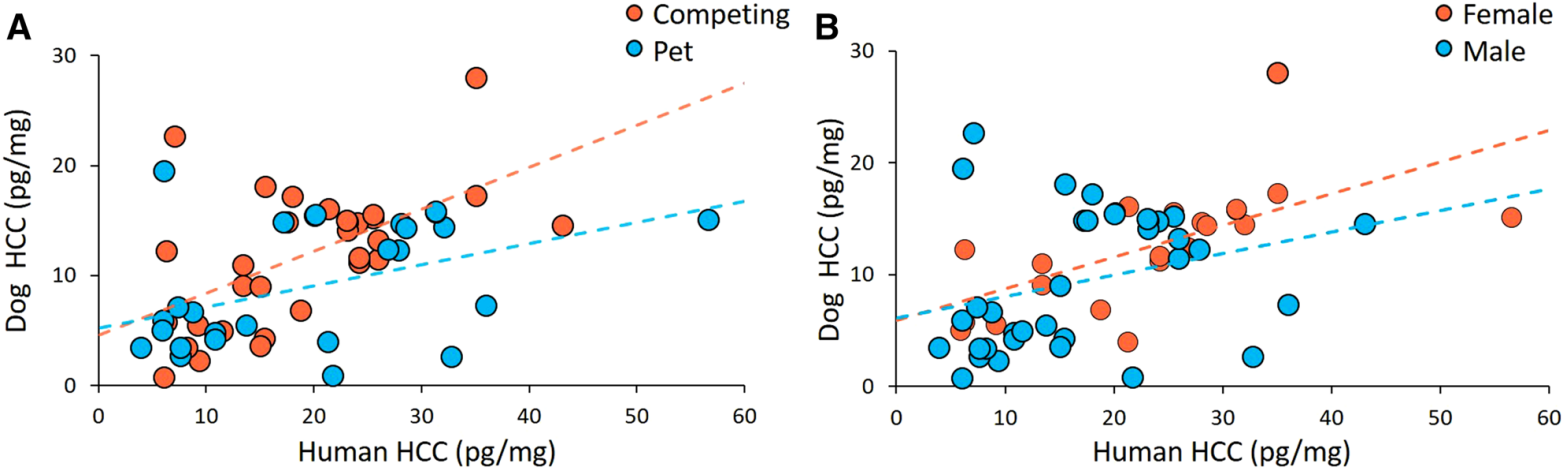
The hair cortisol concentration (HCC) synchronization of dogs and their owners was moderated by lifestyle (**A** competing dogs red, pet dogs blue) and sex of the dog (**B** females red, males blue). Dotted lines show linear fitted lines for lifestyle and sex of the dog.

