# Supplementary material for: Long-term stress levels are synchronized in dogs and their owners
Source: Sci Rep. 2019 Jun 6;9:7391. doi: 10.1038/s41598-019-43851-x (PMC6554395; doi:10.1038/s41598-019-43851-x)
Supplement: Supplementary file 1 — Supplementary files [file 41598_2019_43851_MOESM1_ESM.pdf]

S1

Long-term stress levels are synchronized in dogs and their owners

Ann-Sofie Sundman, Enya Van Poucke, AnnCharlotte SvenssonHolm, Åshild Faresjö,
Elvar Theodorsson, Per Jensen, Lina S.V. Roth

S1

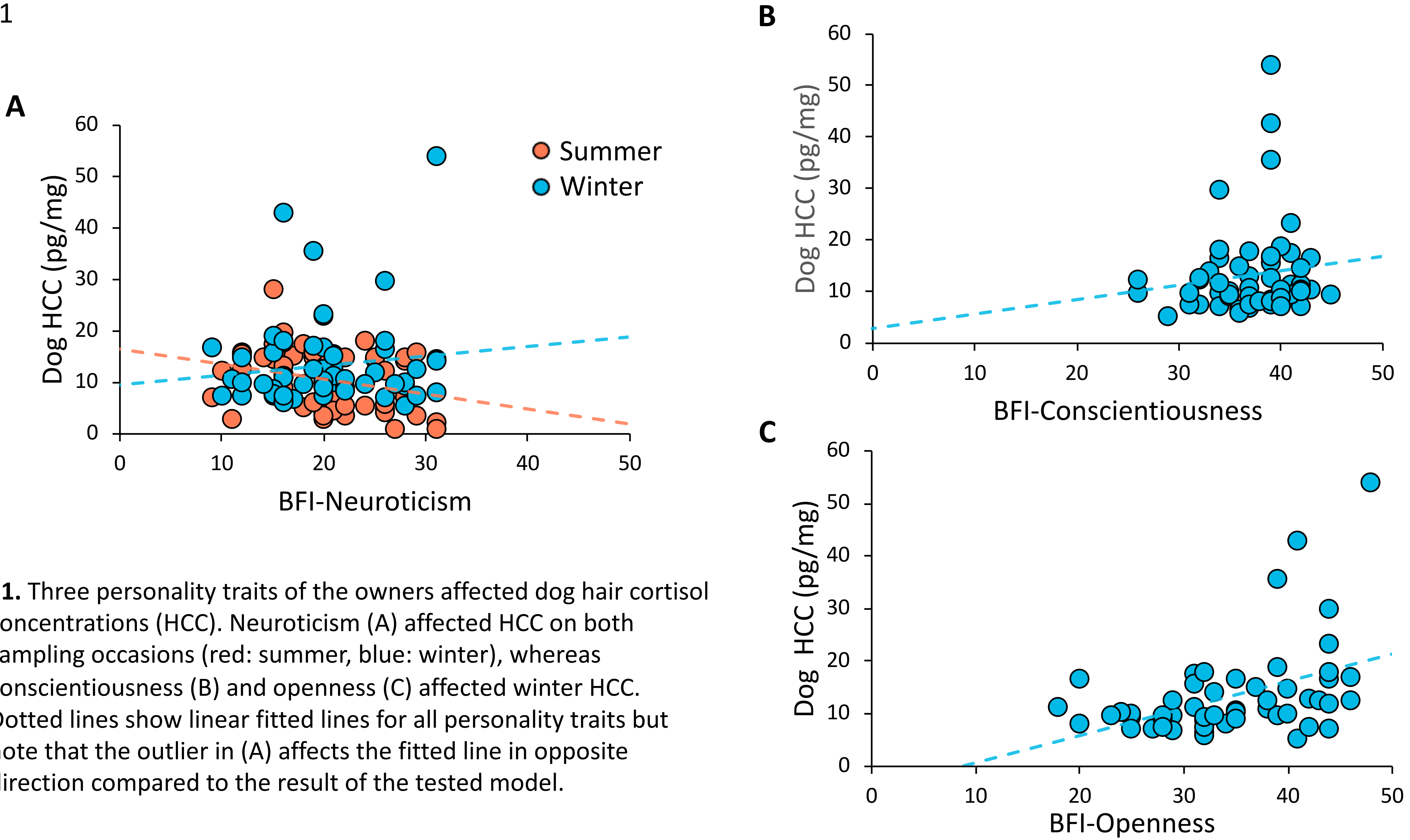

S1. Three personality traits of the owners affected dog hair cortisol concentrations (HCC). Neuroticism (A) affected HCC on both sampling occasions (red: summer, blue: winter), whereas conscientiousness (B) and openness (C) affected winter HCC. Dotted lines show linear fitted lines for all personality traits but note that the outlier in (A) affects the fitted line in opposite direction compared to the result of the tested model.

Ann-Sofie Sundman, Enya Van Poucke, Ann Charlotte Svensson Holm,
Åshild Faresjö, Elvar Theodorsson, Per Jensen, Lina S.V. Roth
Long-term stress levels are synchronized in
dogs and their owners

Table S1 - Final Models

Generalized Linear Model - effents on dogHCC

Dependent variable:	DogHCC summer sample			DogHCC winter sample		
Model type:	Normal with identity			Gamma with log link		
	Wald Chi Square	df	P	Wald Chi Square	df	P
**HumanHCC-summer**	23,697	1	<0.001	13,796	1	<0.001
**Breed**	2,611	1	0,106	6,451	1	0,011
**Sex**	1,678	1	0,195			
**Lifestyle**	0,523	1	0,47			
**HumanHCC*breed**	3,722	1	0,054			
**HumanHCC*sex**	5,2	1	0,023			
**HumanHCC*lifestyle**	6,268	1	0,012			

Generalized Linear Model - owner personality

Dependent variable:	DogHCC summer sample			DogHCC winter sample		
Model type:	Normal with identity			Gamma with log link		
	Wald Chi Square	df	P	Wald Chi Square	df	P
**Sex**	3,765	1	0,052	13,198	1	<0.001
**Extraversion**						
**Agreeableness**				0,945	1	0,331
**Conscientiousness**				15,852	1	<0.001
**Neuroticism**	7,951	1	0,005	4,919	1	0,027
**Openness**	2,907	1	0,088	11,44	1	0,001
**Sex*extraversion**						
**Sex*agreeableness**				4,298	1	0,038
**Sex*conscientiousness**				11,54	1	0,001
**Sex*neuroticism**				21,437	1	<0.001
**Sex*openness**	5,39	1	0,02			

**Generalized Linear Model - dog personality**						
Dependent variable:	**DogHCC summer sample**			**DogHCC winter sample**		
Model type:	Normal with identity			Gamma with log link		
	Wald Chi Square	df	P	Wald Chi Square	df	P
**Sex**	4,042	1	0,044	10,657	1	0,001
**Fearfulness**						
**Aggression towards people**						
**Activity/excitability**						
**Responsiveness to training**				0,533	1	0,465
**Agression towards animals**						
**Sex*Fearfulness**						
**Sex*Aggr. ppl.**						
**Sex*activity/exc.**						
**Sex*responsiveness**				9,144	1	0,002
**Sex*agg. animals**						

**Generalized Linear Mixed Model - seasonal effects**				
Repeated measure:	Season (winter/summer)			
Dependent variable:	DogHCC			
Model type:	Gamma with log link			
	F	df1	df2	P
**Season**	8,706	1	106	0,004
**Breed**				
**Sex**	5,27	1	106	0,024
**Lifestyle**	1,064	1	106	0,305
**Season*breed**				
**Season*sex**				
**Season*lifestyle**	6,143	1	106	0,015

Long-term stress levels are synchronized in dogs and their owners

Ann-Sofie Sundman, Enya Van Poucke, AnnCharlotte SvenssonHolm, Åshild Faresjö,
Elvar Theodorsson, Per Jensen, Lina S.V. Roth

Supplementary Dataset

ID-Number	Breed (SS/BC)	Sex of the dog	Owner Age	Dog Age	Other Animals (N0_Dog1_Cat2_DogCat3)
1	SS	F	66	2	0
2	BC	M	44	10	3
3	BC	M	49	7	1
5	BC	M	34	5	3
7	SS	F	39	9	1
8	SS	M	67	2	2
11	BC	F	33	3	1
12	SS	M	55	4	1
13	SS	M	20	4	0
14	BC	M	68	10	0
15	SS	F	53	5	0
17	BC	F	28	3	3
19	BC	F	59	3	2
22	BC	M	61	2	3
23	SS	F	62	1	2
25	SS	M	27	5	3
26	BC	M	27	2	1
29	SS	M	28	2	1
30	BC	M	62	3	3
31	SS	M	61	8	3
32	BC	F	44	1	3
33	BC	M	42	1	3
34	BC	F	22	1	0
35	SS	M	51	1	3
36	SS	M	51	4	1
37	BC	F	52	9	2
38	BC	F	51	6	3
40	SS	M	58	3	0
41	SS	M	30	3	1
44	SS	M	28	2	0
48	SS	F	28	2	3
49	SS	M	45	5	1
51	SS	M	57	10	3
54	SS	M	49	7	1
55	SS	M	25	8	1
56	BC	M	55	10	
10A	SS	F	39	8	3
10B	SS	F	39	6	3
18A	BC	M	38	8	1
18B	BC	M	38	4	1
20A	BC	F	55	7	1
20B	BC	F	55	3	1
21A	SS	M	34	2	1
21B	SS	M	34	6	1
24A	SS	M	59	8	1
24B	SS	M	59	1	1
28A	BC	F	27	2	1
28B	BC	F	27	4	1
42A	SS	M	62	6	1
42B	BC	M	62	4	1
4A	SS	F	48	10	1
4B	SS	F	48	7	1
50A	SS	M	54	2	1
50B	SS	M	54	1	1
53A	BC	F	56	8	1
53B	BC	F	56	6	1
9A	SS	F	50	4	3
9B	SS	M	50	2	3

SS= Shetland sheepdog
BC= Border collie

ID-Number	Garden (Yes/No)	Lifestyle (Competing/Pet)	Trainings/Week	Competed last 6 months (Yes/No)
1	Y	P	0	N
2	Y	C	3	Y
3	N	C	4,5	Y
5	Y	C	1	Y
7	Y	C	1	Y
8	Y	P	0	N
11	Y	C	5	Y
12	Y	C	3,5	Y
13	N	P	2	N
14	Y	P	0	N
15	Y	P	0	N
17	Y	P	0	N
19	N	P	6,5	N
22	Y	C	3,5	Y
23	Y	P	0	N
25	Y	C	5	Y
26	N	C	3	Y
29	Y	C	5	Y
30	Y	C	6	Y
31	Y	P	1	N
32	Y	P	0	N
33	Y	P	0	N
34	N	P	2	N
35	Y	P	2	N
36	Y	C	1,5	Y
37	Y	P	0	N
38	Y	P	7	N
40	Y	C	2,5	Y
41	N	P	2	N
44	N	P	1	N
48	Y	C	3	Y
49	Y	C	7	Y
51	N	P	0	N
54	Y	P	1	N
55	N	C	5	Y
56		P		
10A	Y	C	1	Y
10B	Y	C	1	Y
18A	Y	P	4	N
18B	Y	P	4	N
20A	Y	C	5	N
20B	Y	C	4	N
21A	N	C	3,5	Y
21B	N	C	3,5	Y
24A	Y	C	5	Y
24B	Y	C	6	Y
28A	Y	C	3	Y
28B	Y	C	4	Y
42A	Y	C	3	Y
42B	Y	C	3,5	Y
4A	Y	P	1	N
4B	N	P	1	N
50A	Y	P	0	N
50B	Y	P	0	N
53A	Y	C	5	Y
53B	Y	C	5	Y
9A	Y	C	2,5	Y
9B	Y	C	2,5	Y

Hair cortisol concentrations (HCC)

ID-Number	Owner Summer H	Dog Summer HCC	Owner Winter HCC	Dog Winter HCC
1	28,2	14,6	24,1	35,4
2	43,1	14,5	78,8	15,6
3	15,6	18,0	12,0	9,4
5	17,6	14,8	5,8	9,4
7	18,9	6,8	12,1	16,4
8	13,8	5,4	13,6	9,6
11	21,5	16,0	33,2	16,6
12	20,1	15,4	2,1	12,7
13	7,5	7,0	7,7	5,3
14	32,9	2,6	19,2	10,4
15	26,9	12,3	18,0	7,1
17	6,0	4,9	15,1	9,7
19	56,7	15,0	29,7	10,7
22	7,2	22,6	28,7	23,2
23	32,1	14,4	26,7	53,9
25	23,1	14,9	26,9	6,7
26	9,4	2,2	19,1	13,9
29	8,3	3,3	16,3	8,9
30	6,1	0,7	18,7	9,4
31	36,1	7,2	44,8	18,7
32	20,2	15,5	49,8	12,3
33	8,8	6,6	4,1	11,1
34	21,4	3,9	6,1	7,3
35	6,2	19,5	13,0	42,7
36	24,1	14,7	7,8	10,3
37				
38	28,6	14,3		
40	15,5	4,2	9,1	14,8
41	17,3	14,8	12,6	11,7
44	21,8	0,8	22,2	8,0
48	9,3	5,4	8,7	8,1
49	18,1	17,2	9,0	9,3
51	4,0	3,4	8,2	7,3
54	27,9	12,2	13,8	7,1
55	11,6	4,9		
56	6,1	5,8	7,5	12,4
10A	13,5	9,0	10,0	11,2
10B	13,5	10,9	10,0	17,4
18A	10,9	4,7	27,2	16,4
18B	10,9	4,1	27,2	6,9
20A	35,1	27,9	6,0	8,5
20B	35,1	17,2	6,0	7,5
21A	15,1	8,9	19,0	9,7
21B	15,1	3,5	19,0	12,3
24A	23,2	14,0	15,1	9,1
24B	23,2	14,8	15,1	9,8
28A	24,3	11,1	12,1	6,4
28B	24,3	11,6	12,1	5,8
42A	26,0	11,4	14,7	17,8
42B	26,0	13,2	14,7	7,3
4A	31,4	15,8	26,6	12,5
4B	31,4	15,7	26,6	16,9
50A	7,7	2,6	8,5	10,2
50B	7,7	3,4	8,5	8,8
53A	6,4	5,7	70,0	17,9
53B	6,4	12,2	70,0	29,7
9A	25,6	15,5	22,0	9,9
9B	25,6	15,1	22,0	14,5

Activity smart collar - PetPace™

ID-Number	Rest %	Low %	Medium %	High %
1				
2	79,9	7,8	8,3	3,9
3				
5	86,3	3,1	5,0	5,5
7	79,2	6,4	10,9	3,5
8	75,5	6,9	10,4	7,2
11	77,4	6,4	13,0	3,2
12	76,4	7,7	12,2	3,7
13	77,0	6,2	10,1	6,7
14				
15	74,5	8,6	9,2	7,7
17	76,2	5,6	10,0	8,2
19				
22	77,0	6,9	11,1	4,9
23	77,5	7,7	11,7	3,1
25	66,4	9,4	16,7	7,5
26	80,5	5,9	11,1	2,6
29				
30	78,1	6,0	8,5	7,3
31	84,1	5,9	8,4	1,6
32	75,0	6,4	13,6	5,0
33				
34	74,8	7,0	10,9	7,3
35				
36	73,4	5,9	14,4	6,3
37	73,1	8,1	11,8	7,0
38	80,7	5,1	9,8	4,4
40	64,2	9,4	18,1	8,2
41	81,5	7,5	8,6	2,4
44	65,5	10,4	19,6	4,5
48	69,3	9,9	14,9	6,0
49	78,9	5,9	9,6	5,6
51	78,1	6,5	6,6	8,9
54	78,8	9,2	9,7	2,3
55				
56	83,0	5,6	8,6	2,8
10A	67,8	10,9	19,3	2,1
10B	75,6	7,6	12,7	4,2
18A	84,8	4,4	5,2	5,6
18B	83,3	4,2	6,5	6,1
20A	87,4	4,6	6,6	1,5
20B	83,8	6,0	6,9	3,3
21A	72,0	8,6	14,6	4,8
21B	78,3	6,9	9,5	5,2
24A	75,4	7,3	10,0	7,3
24B	62,0	8,1	18,3	11,7
28A	77,1	8,0	8,4	6,4
28B	71,7	9,7	13,8	4,7
42A				
42B				
4A				
4B				
50A				
50B	60,1	10,6	22,1	7,2
53A	79,3	6,9	9,0	4,9
53B	79,3	7,5	9,9	3,3
9A	83,1	5,1	9,9	2,0
9B	80,3	6,3	10,8	2,6

Personality traits

Owner

ID-Number	BFI-Extraversion	BFI-Agreeableness	BFI-Conscientiousness	BFI-Neuroticism	BFI-Openness
1	34	44	39	19	39
2	31	38	39	15	31
3	31	33	35	24	28
5	33	40	45	14	23
7	32	41	43	9	20
8	20	35	31	24	33
11	31	35	39	20	35
12	28	36	37	21	42
13	13	36	29	28	41
14	33	42	43	11	35
15	34	41	42	12	27
17	25	42	34	21	33
19	26	34	37	16	38
22	19	38	41	20	44
23	25	37	39	31	48
25	33	37	37	17	29
26	28	32	33	31	33
29	22	37	37	22	28
30	32	38	41	27	39
31	31	37	40	15	39
32	29	39	32	29	43
33	21	31	42	21	18
34	32	40	32	20	28
35	30	38	39	16	41
36	21	32	42	22	24
37	39	38	36	23	47
38	17	31	35	25	47
40	33	44	36	21	37
41	23	37	34	25	44
44	35	43	38	31	34
48	31	29	39	22	20
49	33	40	41	18	32
51	23	40	31	29	42
54	29	31	40	10	25
55	33	33	42	18	36
56	32	37	32	19	38
10A	31	41	41	16	31
10B	31	41	41	16	31
18A	27	29	34	26	44
18B	27	29	34	26	44
20A	34	43	39	15	32
20B	34	43	39	15	32
21A	28	38	26	20	29
21B	28	38	26	20	29
24A	26	26	35	28	25
24B	26	26	35	28	25
28A	27	38	36	16	32
28B	27	38	36	16	32
42A	25	38	37	16	32
42B	25	38	37	16	32
4A	33	32	39	19	46
4B	33	32	39	19	46
50A	32	38	40	20	35
50B	32	38	40	20	35
53A	33	38	34	26	44
53B	33	38	34	26	44
9A	26	43	42	12	40
9B	26	43	42	12	40

Personality traits

Dog

ID-Number	Factor1 Fearfu	Factor2 Aggre	Factor3 Activity E	Factor4 Respo	Factor5 Aggre
1	28	10	117	66	39
2	49	28	127	63	54
3	39	16	119	58	40
5	56	10	101	66	26
7	43	17	85	54	44
8	33	10	123	51	38
11	37	10	103	56	25
12	43	14	117	60	43
13	58	12	132	54	33
14	52	16	114	67	63
15	92	10	81	67	37
17	46	10	96	53	46
19	31	10	104	67	18
22	46	10	120	65	22
23	63	18	112	39	41
25	76	27	94	59	38
26	44	10	126	59	22
29	49	10	100	64	26
30	38	13	123	60	41
31	24	10	107	67	22
32	56	17	108	56	29
33	47	13	124	61	25
34	60	11	112	52	41
35	50	11	120	64	36
36	38	13	96	47	71
37	54	14	103	65	40
38	44	16	106	62	42
40	72	16	98	59	23
41	103	17	100	53	51
44	48	11	118	37	45
48	59	12	99	56	39
49	51	10	117	67	31
51	67	10	92	67	28
54	36	15	100	43	69
55	66	23	97	65	42
56					
10A	48	14	131	37	50
10B	45	15	120	44	68
18A	26	10	101	52	27
18B	85	17	125	54	62
20A	47	14	121	66	31
20B	57	10	123	60	22
21A	55	26	116	38	46
21B	63	23	75	57	50
24A	45	13	64	56	43
24B	49	14	123	41	54
28A	75	10	103	60	24
28B	44	16	102	68	41
42A	69	20	94	49	53
42B	48	14	88	62	55
4A	38	13	92	47	37
4B	67	24	95	47	44
50A	43	10	89	64	27
50B	113	10	126	27	63
53A	27	16	114	63	21
53B	57	10	101	55	31
9A	41	10	119	69	30
9B	69	10	91	63	23
